# Distinct Mechanisms Regulating Gene Expression Coexist within the Fermentative Pathways in *Chlamydomonas reinhardtii*

**DOI:** 10.1100/2012/565047

**Published:** 2012-06-18

**Authors:** Larisa Angela Swirsky Whitney, Giacomo Novi, Pierdomenico Perata, Elena Loreti

**Affiliations:** ^1^Department of Crop Plant Biology, University of Pisa, Via Mariscoglio 34, 56124 Pisa, Italy; ^2^PlantLab, Institute of Life Sciences, Scuola Superiore Sant'Anna, Via Mariscoglio 34, 56124 Pisa, Italy; ^3^Institute of Agricultural Biology and Biotechnology, National Research Council, Via Moruzzi 1, 56100 Pisa, Italy

## Abstract

Under dark anoxia, the unicellular green algae *Chlamydomonas reinhardtii* may produce hydrogen by means of its hydrogenase enzymes, in particular HYD1, using reductants derived from the degradation of intercellular carbon stores. Other enzymes belonging to the fermentative pathways compete for the same reductants. A complete understanding of the mechanisms determining the activation of one pathway rather than another will help us engineer *Chlamydomonas* for fermentative metabolite production, including hydrogen. We examined the expression pattern of the fermentative genes *PDC3, LDH1, ADH2, PFL1*, and *PFR1* in response to day-night cycles, continuous light, continuous darkness, and low or high oxygen availability, which are all conditions that vary on a regular basis in *Chlamydomonas*' natural environment. We found that all genes except *PFL1* show daily fluctuations in expression, and that *PFR1* differentiated itself from the others in that it is clearly responsive to low oxygen, where as *PDC3, LDH1*, and *ADH2* are primarily under diurnal regulation. Our results provide evidence that there exist at least three different regulatory mechanisms within the fermentative pathways and suggest that the fermentative pathways are not redundant but rather that availability of a variety of pathways allows for a differential metabolic response to different environmental conditions.

## 1. Introduction

Interest in the unicellular green alga *Chlamydomonas reinhardtii* (referred to here as *Chlamydomonas* throughout) has increased over the past decade in the hope that it may one day be possible to harness its exceptional capacity to produce hydrogen. Hydrogen may be used as a renewable energy carrier whose combustion does not release CO_2_ into the atmosphere, rendering it more attractive than other potential renewable energies such as ethanol [1].

*Chlamydomonas* synthesizes hydrogen in a reaction catalyzed mainly by its hydrogenase1 (HYD1) enzyme [2]. Due to the hydrogenases' extreme sensitivity to oxygen [3], sustained H_2_ synthesis occurs only under anoxia. In its natural environment, *Chlamydomonas* may be subjected to hypoxia or anoxia on a daily basis after sunset, when the absence of photosynthesis coincides with high rates of respiration [4]. In the lab, low oxygen cultures may also be obtained in the light in cases where photosynthetic O_2_ evolution does not cover respiratory requirements, for example, by anaerobic gas influxation into liquid cultures or a number hours after being subjected to sulfur starvation [5, 6]. A number of studies have shown that *Chlamydomonas* acclimates to anoxia by changing its metabolism from aerobic to fermentative [7–11].

 In dark anoxia the source of electrons for the hydrogenases must come from the degradation of intercellular carbon stores, probably starch [7] as *Chlamydomonas *does not appear to assimilate extracellular sugars. In this path, starch is broken into glucose and metabolized to pyruvate via glycolysis, which is then converted to acetyl-CoA by either pyruvate-formate lyase (PFL1) or pyruvate-ferredoxin oxidoreductase (PFR1). One molecule of CO_2_ is released in the PFR1 reaction, and ferredoxin is reduced. It is ferredoxin which passes electrons to the hydrogenase [12]. The acetyl-CoA generated may be further metabolized to acetic acid in two steps by phosphate acetyl-transferase (PAT1 or PAT2) and acetate kinase (ACK1 or ACK2) generating an ATP [11, 13]. Alternatively, acetyl-CoA may be converted to ethanol by alcohol dehydrogenase1 (ADH1) reoxidizing two NADH and thereby allowing glycolysis to continue [14].

 In *Chlamydomonas*, in addition to these H_2_/CO_2_/acetate/ethanol-generating pathways, a number of other fermentative pathways leading to a range of products are present, among which, malate, lactate, and succinate [13, 15, 16]. The relative production of each metabolite depends on culture conditions [7, 17]. The capability of *Chlamydomonas* to vary its fermentation profile is known as “flexibility”, and it is of relevance for the development of strategies to engineer microorganisms for renewable energy production [18]. However we still lack a detailed understanding of factors determining relative contribution of each pathway in response to stress and the role of each pathway in sustaining cell viability during anaerobiosis.

 A number of factors that may function as switch between pathways have been suggested. Acidification of the cytoplasm, a characteristic feature of the response of many plant tissues to oxygen deprivation [19, 20] activates pyruvate decarboxylase (PDC) and inhibits lactate dehydrogenase (LDH) in higher plants. The cell thereby reduces lactate production and redirects the metabolism towards ethanol fermentation [21]. The degree of anoxia may be another potential path switch determinant. The proportion ethanol : acetate generated has been shown to depend upon oxygen tension [22, 23]. Different time points following anoxia induction may show different metabolic profiles. Such is the case when anoxia is induced by sulfur starvation in the light [16].

The activity of each pathway may influence the activity of the others in a type of cross-regulation [24] and this phenomenon is beginning to be studied by selectively blocking individual pathways using mutants or pharmaceuticals. A hydEF-deficient strain which produces no functional hydrogenase enzymes activated a new succinate fermentation pathway under anoxia which was not present in the background strain instead of upregulating a preexistent pathway [13]. A strain with an insertional mutation in the *ADH1* gene which does not produce any ethanol in anoxia and in which no ADH1 protein can be detected also upregulates a new pathway, the synthesis and extracellular accumulation of glycerol, not present in the background strain [14]. A mutant strain unable to synthesize the PFL1 protein secretes no formate but produces more ethanol, D-lactate, and CO_2_ than the wild type [24]. Interestingly, reduced levels of *HYD1* transcript and HYD1 protein were found in *pfl1* [24]. This was unexpected as PFL1 may be viewed as a competitor for electrons with HYD1, such that its elimination should have forced more electrons through PFR1 and on to the hydrogenase. It was suggested that the metabolite produced by PFL1, formate, could play a role in gene regulation and influence the ratio of fermentative products [24]. In *Chlamydomonas* the effects of formate have not been determined, but the *plf1* mutant provides us with further insight into the complexity of the regulation of fermentative metabolism and proves that the activation/deactivation of one pathway does not necessarily influence metabolic fluxes the way we expect.

 Light availability (or absence) is also of relevance. Diverse conditions of light and dark affect starch degradation, fermentative gene regulation and enzyme activity [7, 13, 24–26], for example, the *PFL1* gene is not upregulated in response to anoxia under light to the same extent as it is in response to dark anoxia [23]. Light is an input which regulates which “sets the time” of the circadian clock. In this context it is interesting to note that *Chlamydomonas* shows diurnal variation of intercellular starch reserves, with a peak starch content in the middle of the night [27]. A number of genes involved in carbohydrate metabolism and fermentation were found to be under circadian control including D-lactate dehydrogenase [28], a CoA-linked acetaldehyde dehydrogenase and iron-dependent alcohol dehydrogenase (*ADH1*), a ferredoxin and the hydrogenase (*HYD2*) gene itself [26, 28].

 In this work we examine the pattern of expression of selected fermentative gene in synchronous cells in a photoperiod and in response to continuous light/dark. Also, since the oxygen levels in the media of liquid cultures of *Chlamydomonas* depend on light conditions, we examine whether or not the observed patterns are determined by changes in oxygen levels. We uncovered the existence of three different gene expression profiles within the fermentative pathways, which might be an indication of a differential adaptive response of this green algae to different environmental inputs or changing metabolic factors which can coexist with low oxygen stress.

## 2. Materials and Methods

### 2.1. Strains and Growth Conditions

The *Chlamydomonas reinhardtii* 11-32c wild-type strain was obtained from the algae collection of the Gottingen University, Germany. Cells were grown in Tris acetate-phosphate (TAP) medium (pH 7.2) as described by Harris [29]. Cell suspensions were grown to a concentration of 1 × 10^6^ cells mL^−1^. Synchronous *Chlamydomonas* cell cultures were obtained by alternating light (12 h) and dark (12 h) periods for 6 days and maintained by daily dilution of the cultures to a starting density of cultures 10^6^ cells mL^−1^. Cell division was visually monitored using a microscope and by counting cells with a Bürker chamber. The light intensity during the light period was 70 *μ*mol m^−2^ s^−1^. During 64 h long experiments, samples were collected every 4 h and corresponding volume of fresh medium added. For the continuous darkness or light experiments, the culture was divided and one part was transferred to continuous darkness, one part was transferred to continuous light at the intensity given earlier, and one part was left in a 12 h : 12 h light : dark photoperiod.

The experiments described in Figure 3 were performed by continuously fluxing 1% oxygen or air (21% oxygen) in the flasks containing the cultures in a 12 h : 12 h light : dark photoperiod.

### 2.2. RNA Extraction and qPCR

Cultures of *Chlamydomonas* 1 × 10^6^ cells/mL^−1^ were pelleted at 4,000 g for 1 min. The pellet was resuspended in the following buffer diluted 1 : 1 with water: 2% SDS, 400 mM EDTA, 100 mM Tris-HCl (pH 8.0). The resulting solution was extracted once with phenol-chloroform 1 : 1 (v/v) supplemented with sodium-acetate 0.3 M (pH 5.0). The samples were vortexed briefly and centrifuged at 12,000 g for 10 min. The supernatant was extracted twice with phenol-chloroform (without sodium acetate), then extracted a final time in chloroform. Samples were precipitated using LiCl 8 M added 1 : 1 (v : v) for 4 h at 4°C, centrifuged at 13000 g and the pellet washed in 70% ethanol and finally resuspended in DEPC water. RNA quality was checked on 1% agarose gel, and quantified with spectrophotometric readings. RNA was subjected to a DNAse treatment using a TURBO DNA-free kit (Ambion, Inc., Austin, TX, USA). One microgram of each sample was reverse transcribed into cDNA with an iScript cDNA Synthesis kit (Bio-Rad). Real-time reverse-transcription polymerase chain reaction amplification was carried out with an ABI Prism 7000 sequence detection system (Applied Biosystems, Foster City, CA, USA), using the ribosomal protein L13 (RPL13) RNA as an house-keeping control gene, its expression was not affected by our treatments. The primers used were as follows: *ribosomal protein L13* (RPL13 fw AGCACGGCTAGAGACAGATG; RPL13 rev TAGTGCGTGGCTGTTTGTTG); *alcohol dehydrogenase 2* (ADH2 fw GGAGATCCTGGAGTTCAAGC; ADH2 rev CAGGGCACTCATACATCAGC); *pyruvate decarboxylase 3* (PDC3 fw CTGTGCGTGACCTTCTGTGT; PDC3 rev CTGTGCGTGACCTTCTGTGT); *pyruvate formate lyase 1* (PFL1 fw CCGTTGGACTATGAGGAGGT; PFL1 rev GCCGCTCGTAGTCGTACTTG); *pyruvate feredoxin oxidoreductase *(PFR1 fw CAGCAACCTGGTGGTGTTC; PFR rev GGTGATGGGGTAGATGAAGG); *hydrogenase 1 *(HYD1 fw GGGAACGTGGGTAGCATTTA; HYD1 rev ACACCAACGTCAATCGCATA); *lactate dehydrogenase 1* (LDH1 fw GAAGATGAAGAAGGGCGTCA; LDH1 rev CCGCCCTCATCTCATACAC).

 A cDNA pool of all samples was analysed for a standard dilution series to monitor the qPCR efficiency for each primer pair. The PCR reactions were carried out using 40 ng of cDNA and iQ SYBR Green SuperMix (Bio-Rad Laboratories Inc.) following the manufacturer's protocol. Relative quantification of each mRNA was performed using the comparative CT method, as described by the manufacturer (ABI PRISM 7700 Sequence Detection System User Bulletin no. 2; Applied Biosystems).

## 3. Results

### 3.1. Daily Expression Profiles Differ in Different Branches of the Fermentative Pathway

**Figure 1:**
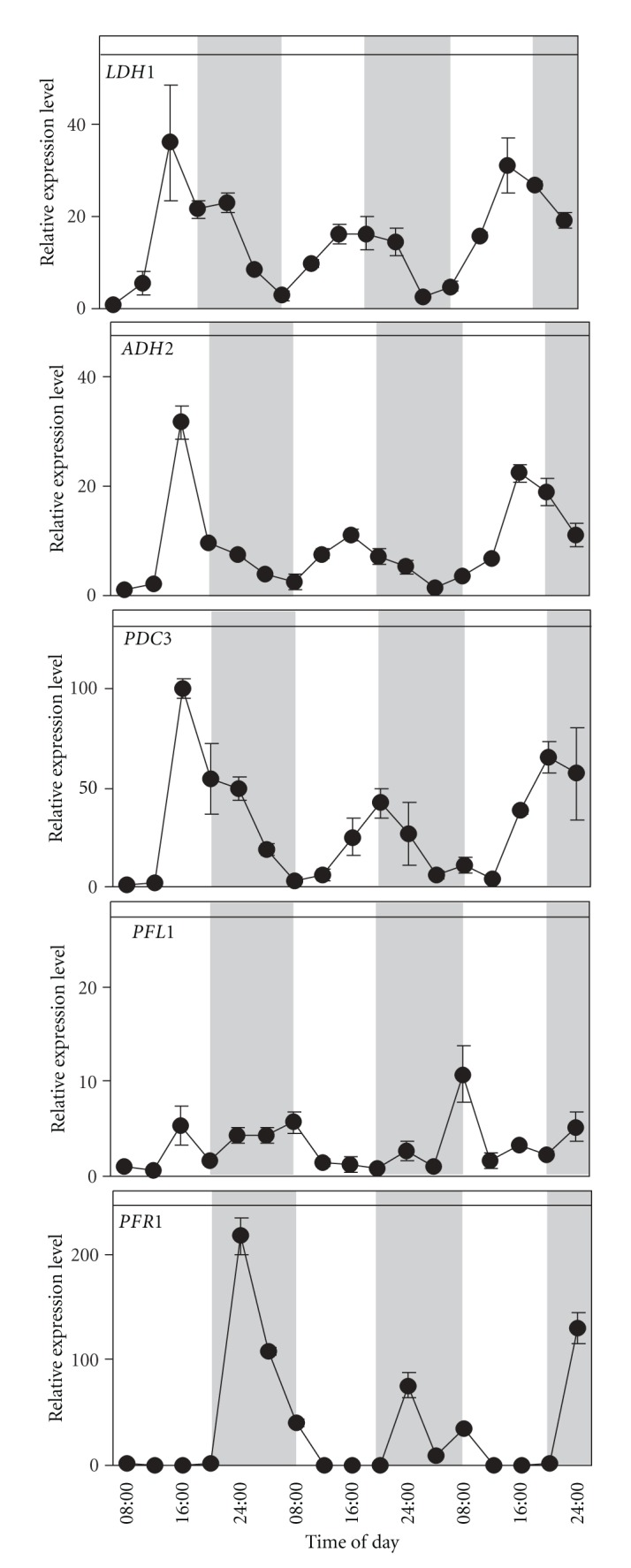
Expression patterns for *LDH1, ADH2, PDC3, PFL1,* and *PFR1* in synchronized *Chlamydomonas* cells over 3 cycles of day and night in control conditions (12 h : 12 h light : dark photoperiod; 23°C), with samples taken every 4 hours. Relative expression levels were measured by real-time reverse-transcription polymerase chain reaction (qPCR) (1 = the value of expression measured at the first data point which corresponds to 8:00 on the first day). Data are mean ± SD, *n* = 3 replicates. When not shown, the error bars were smaller than the symbols.

Gene expression profiles were first analysed in conditions simulating those found in a *Chlamydomonas*' natural environment, in a 12 : 12 hour light : dark photoperiod (Figure 1). Samples taken every 4 hours for 64 hours revealed that *LDH1, ADH2*, and *PDC3*, showed very similar expression patterns. All three genes showed the lowest expression level early in the morning, but a peak in the late afternoon between 4:00 pm and 8:00 pm, decreasing their expression gradually during the night phase (Figure 1). *ADH2* and *PDC3* genes, on the path to ethanol, showed a similar pattern as *LDH1*. Their pattern contrasted sharply with that of the gene on the path to hydrogen production, *PFR1*, which showed a peak in expression soon after the start of the night phase, then decreasing gradually during the night hours. *PFR1* expression was virtually zero throughout the light phase (Figure 1). While the aforementioned genes followed a well-defined albeit contrasting pattern, *PFL1*, which belongs to a different branch of the fermentative pathways, did not seem to follow a clear pattern (Figure 1). The maximum amplitude of its oscillations was not more than 4-fold where as compared to the 30–200-fold amplitudes were observed for the above mentioned genes, and the oscillations seemed to be random. It is likely therefore that the peaks observed for this gene are baseline variations.

### 3.2. Genes in the Fermentative Pathway Respond Differently to Continuous Light and Continuous Dark

**Figure 2:**
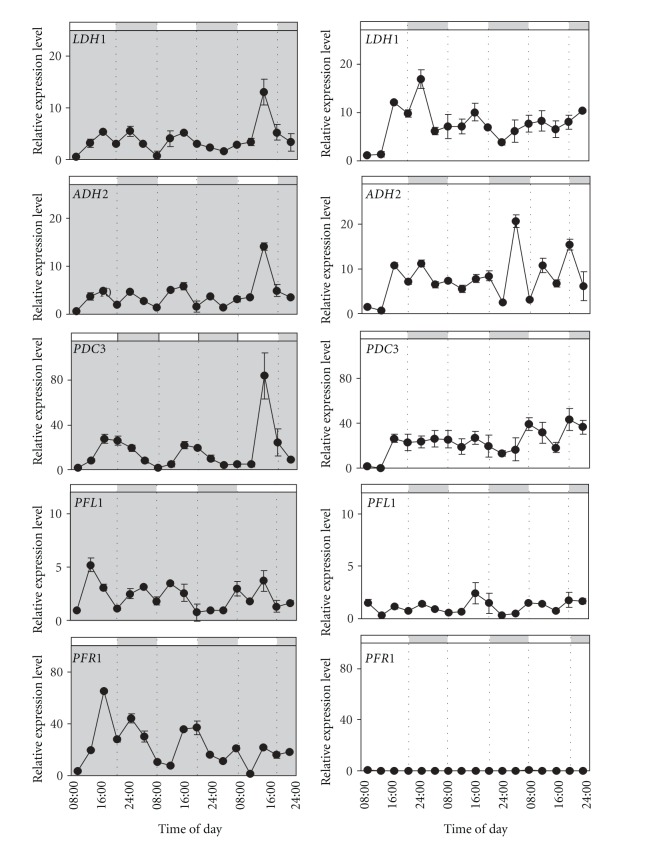
Expression patterns for *LDH1, ADH2, PDC3, PFL1,* and *PFR1* in synchronized *Chlamydomonas* cells over 24-hour cycles are shown in continuous dark (left, shaded box) and continuous light (right, white box), with samples taken every 4 hours. Cells were grown at 23°C. The bar on top of each graph depicts the corresponding virtual day/night periods. Relative expression levels were measured by real-time reverse-transcription polymerase chain reaction (qPCR) (1 = the value of expression measured at the first data point which corresponds to 8:00 on the first day). Data are mean ± SD, *n* = 3 replicates. When not shown the error bars were smaller than the symbols.

We wished to find out if the clear expression pattern shown in a 12 h : 12 h photoperiod by four of the five genes examined would be maintained under continuous light or dark. Also we were curious of the response of *PFL1 *to continuous light and dark, the only gene which had not shown a clear pattern of expression in a photoperiod. As before, samples were taken every 4 hours for 64 hours. Out of all the genes examined, *PDC3* maintained a near perfect oscillation pattern in continuous dark, similar to that observed in a photoperiod (Figure 2). *ADH2* continued to show oscillation though it seems that the virtual day time peak was lowered, and a small dark phase peak was evidenced, highlighting the existence of a double-peak for *ADH2* in continuous dark (Figure 2). It cannot be excluded that a small dark phase peak also exists for *ADH2* when cells are grown in a photoperiod, it may simply be less visible due to the high late afternoon peak which is present under these conditions (Figure 1). The *LDH1* the gene responsible for lactate production maintained a moderate fluctuation in continuous dark, though from our results the amplitude seems to be reduced when compared to the clear fluctuations observed in a photoperiod and the pattern becomes less and less clear after 48 hours of continuous dark (Figures 1 and 2). *LDH1, ADH2*, and *PDC3* showed a similar response to continuous light in that they lose their clear fluctuation pattern and remain expressed at a medium-high level (Figure 2). Taken together, these observations suggest that *LDH1, ADH2*, and *PDC3* respond to two factors. A circadian input is certainly present, but this signal may be disrupted in the presence of continuous light. Results for *PFL1* both in continuous dark and continuous light coincide with the results obtained for *PFL1* in a photoperiod, and, that is, *PFL1* expression seems unrelated to diurnal variation. In continuous dark *PFL1* shows some fluctuation in expression, however the amplitude is low, not more than 5 fold, and irregular (Figure 2). In continuous light, *PFL1* expression is lower than that observed in continuous dark (Figure 2). *PFR1* clearly differentiates itself from the other genes in that it maintains fluctuations in continuous dark, though less regular than those observed in a photoperiod, while in continuous light *PFR1* expression is reduced nearly to zero.

### 3.3. Light/Oxygen Responsiveness of Genes in the Fermentative Pathway

**Figure 3:**
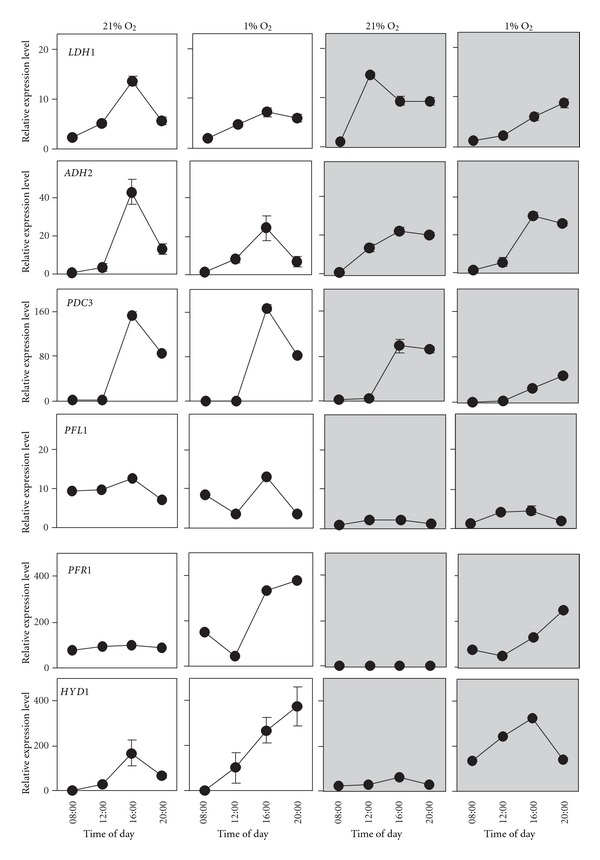
The combined effect of 21% versus 1% O_2_ influxation in the culture media and light versus dark on the expression of *LDH1, ADH2, PDC3, PFL1,* and *PFR1* followed a 12-hour period starting at 8:00 in the morning, with samples taken every 4 hours. Relative expression levels were measured by real-time reverse-transcription polymerase chain reaction (qPCR) (1 = the lowest value of expression measured in the 4 conditions). Data are mean ± SD, *n* = 3 replicates. When not shown, the error bars were smaller than the symbols.

In a photoperiod, oxygen in the culture media of synchronous cells is high during the day and low during night phase [26]. We were curious to see how the fluctuations observed would respond if the level of oxygen in the media were controlled by influxing hypoxic gas or air. Also we wanted to check if the expression pattern of the genes could be influenced by the presence of light and dark under contrasting oxygen availability. Synchronous cells were treated with an influx of N_2_ gas containing 1% O_2_ were confronted with synchronous cells influxed with air (=21% O_2_) both in the light and in the dark. Sampling began at 8:00 in the morning and was done every 4 hours until 20:00. Once again, the *LDH1, ADH2*, and *PDC3* showed similar responses (Figure 3). In the presence of high oxygen and light, *LDH1* showed a normal peak (similar to what was observed in a photoperiod, Figure 1). In response to high oxygen in the dark, a peak in expression was still present during the day, but it was anticipated (Figure 3). In the presence of low oxygen (both in the light and in the dark), the peak was slightly lower or, perhaps, delayed. We conclude that the daily fluctuations in expression observed for *LDH1* are present independently of the concentration of oxygen or the presence of light/dark. The level of oxygen and light seems to modulate the pattern of the fluctuations without eliminating them. *ADH2*, on the path to ethanol, did not seem particularly affected by any of the oxygen treatments, apart from a lower peak of expression in the light in combination with low oxygen, as also observed for *LDH1* (Figure 3). *PDC3* expression was not affected at all by oxygen in the light, while it was influenced by the combined presence of low oxygen and dark (Figure 3). Under these conditions the peak was lowered, or, perhaps here as well, delayed. None of the treatments in this experiment had an effect on *PFL1* (Figure 3), as if to confirm what was observed in the previously discussed experiments. *PFR1*, on the path to hydrogen, showed a markedly different response to the treatment. *PFR1* expression was upregulated under low oxygen and eliminated in the presence of high oxygen, independently of light conditions (Figure 3). We were curious to see if the gene directly encoding the enzyme responsible for hydrogen production, *HYD1* [2] would show a similar response oxygen and dark as *PFR1,* as the two genes are part of the same pathway. Indeed such was the case, although *HYD1* expression, while visibly reduced in presence of high oxygen (Figure 3), was not eliminated completely as was observed for *PFR1*.

## 4. Discussion

Traditionally, fermentation is considered to substitute Krebs cycle and the electron transport elements of respiration in cases when oxygen is not available in order to regenerate NAD+ to allow glycolysis continue and produce at least some ATP. For organisms such as *Chlamydomonas*, which possesses a variety of fermentative pathways, a question arises as to which of these pathways will activate in the absence of oxygen, or perhaps will they all?

**Figure 4:**
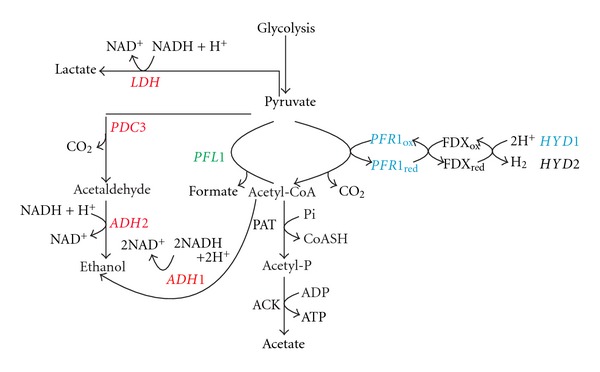
Diagram depicting the fermentative pathways of *Chlamydomonas* split into 3 functional subpaths based on response profile to circadian inputs, light (dark), and oxygen in cells synchronized to a 12 h : 12 h light : dark photoperiod and grown at 23°C. In red: *LDH1, ADH1, ADH2,* and *PDC3* which are under circadian regulation. In green: *PFL1* which did not show a circadian pattern of expression, nor did it respond to changes in light (dark) or oxygen. In blue: *PFR1* and *HYD1* which both show circadian expression patterns and are also responsive to changes in oxygen, but not to changes in light.

 Four of the genes (*LDH1*, *ADH2*, *PDC3*, and *PFR1*) studied in this paper fluctuated on a daily basis (Figure 1), supporting results obtained in previous studies which take into examination the expression patterns of others fermentative genes [26, 28]. However, *LDH1*, *ADH2*, and *PDC3* differentiated themselves from *PFR1* in that the first three show a day-time peak whereas the latter shows a night time peak suggesting that at least two different regulation profiles coexist in two different parts of the fermentative metabolism. *PFL1*, in contrast to the aforementioned genes, did not show any regular daily fluctuations (Figure 1), suggesting the existence of a third regulation profile within the fermentative pathways, or more likely, the insensitivity of *PFL1* to the other two regulatory pathways. The different profiles of gene regulation identified in our experiments are represented in Figure 4. In this paper we investigated some possible causes of these fluctuations, and in particular whether or not changes in light (dark) or low oxygen may influence them. Variations in light and oxygen availability are two regularly changing factors that occur over the day-night period in a natural context.

 The results suggest the existence of a double input in the regulation of *LDH1, ADH2, PDC3*, and *PFR1*: circadian cycle regulation which can be interrupted in continuous light, perhaps due to a loss of cell synchrony [26] as green algae have been reported to lose synchrony within 24 hours following exposure to continuous light [30, 31]. The cell cycle has been shown to be under circadian regulation [32] though cell division is also gated by metabolic criteria (minimal volume and energy content), factors which in turn are influenced by light through photosynthesis [30, 31]. The observed circadian fluctuations in fermentative gene expression might therefore be gated by other factors, among which continuous light due to its disruptive effect on the cell cycle. Interrupted circadian fluctuation of starch accumulation has been reported in *Chlamydomonas* in response to nutrient starvation, a condition which slows or stops the cell cycle [27].

 In photosynthetic organisms there is a tight relationship between light and oxygen availability. We showed that *ADH2* expression is not influenced by light (dark) or oxygen status over a 12-hour period (which is too short a period for cell synchrony to be lost) and that for *LDH1* and *PDC3*, only low oxygen tends to slightly reduce (or perhaps delay) the normal midafternoon peek (Figure 3). Thus, variations in oxygen availability and light (dark), within the range of values used in our experiments (which simulate conditions that might occur in a natural context) do not determine the presence of the fluctuations we observed in the expression of these genes.

*PFR1* expression, contrasting with what was observed for *LDH1, ADH2*, and* PDC3* is completely eliminated in continuous light. Also, our experiments showed that *PFR1* expression is upregulated by low oxygen, irrespective of light conditions (Figure 3). When confronting *PFR1* expression with that of *HYD1*, the main gene responsible for hydrogen production [2], we found that they showed a similar response to combinations of light/dark and low/high oxygen (Figure 3), further supporting the hypothesis the hydrogen producing branch of the fermentative pathway, at least at the mRNA level, is regulated differently than the branches that lead to ethanol and lactate. It is tempting to speculate that *PFR1* expression is under exclusive regulation by low oxygen, and that the absence of expression under light is an indirect consequence of oxygen production by photosynthesis. This would allow a coordinated expression of both *PFR1* and *HYD1* to produce hydrogen exclusively under anoxia.

*PFL1* did not show regular fluctuations in any of the experimental conditions used in this paper suggesting the existence of a third expression profile within the fermentative metabolism. From our results, *PFL1* does not appear to be modulated in response to stresses or environmental inputs at the RNA level. However the presence of a functional PFL1 protein is essential to the normal functioning of the other pathways, as its absence changes the fermentative product ratio and the change is not a simple increase of products produced by enzymes which compete for the same substrate [24]. PFL1 catalyzes a reaction that produces formate as one of its products. Formate is not a neutral metabolite, it has been suggested to play a role in gene regulation, possibly repressing the hydrogen metabolism [24]. The involvement of formate in the hydrogen metabolism has been demonstrated in *Escherichia coli* [33]. In *Chlamydomonas *formate is known to influence phothosyntesis by inhibiting electron and proton transfers in photosystem II [34]. We found that *PFL1* is not influenced by light or oxygen leaving open the possibility of a feedback regulation by the product of its reaction, in support of what was suggested by Philipps et al. [24]. We could hypothesize that this feedback regulation may act to prevent the production of a metabolite which can downregulate of photosynthesis.

Whether variations in RNA prove to be correlated with metabolic outcome or not, the existence of differential regulation in different branches of the path is indicative that different mechanisms are likely at work to determine different types of fermentation. Factors determining switches between fermentative pathways will be of particular interest in engineering *Chlamydomonas* for hydrogen production. Different branches of the pathways leading to different products may determine changes in pH (lactic acid, acetic acid), cause (or limit) accumulation of toxic metabolites (ethanol), or either the reoxidation of NADH over the synthesis of ATP (or vice versa), according to current physiological requirements [21, 23].

 In the natural environment, in addition to changes in light availability and the possible occurrence of low oxygen conditions, organisms may be simultaneously subjected to other stimuli which, above a certain level of intensity or length of time they may be perceived as stresses. Temperature typically varies on a daily basis and provides an input for setting the circadian clock [35]. Internal factors such as cell cycle status, and the circadian “time,” also vary at different times of day. We believe that the precise physiological response to a change in a specific environmental factor will vary according to the status of the other inputs. Therefore, under given circumstances, a particular type of fermentation maybe suitable, but in other conditions, a different fermentative path may be preferable. The possession of numerous fermentative options certainly could be viewed as advantages for a water dwelling yet aerobic organism so easily subjected to low oxygen on a regular basis. Uncovering the precise role of each of these paths will represent an interesting theme for future research.
